# Emerging implications of policies on malaria treatment: genetic changes in the *Pfmdr-1* gene affecting susceptibility to artemether–lumefantrine and artesunate–amodiaquine in Africa

**DOI:** 10.1136/bmjgh-2018-000999

**Published:** 2018-10-19

**Authors:** Lucy C Okell, Lisa Malene Reiter, Lene Sandø Ebbe, Vito Baraka, Donal Bisanzio, Oliver J Watson, Adam Bennett, Robert Verity, Peter Gething, Cally Roper, Michael Alifrangis

**Affiliations:** 1 MRC Centre for Outbreak Analysis and Modelling, Department of Infectious Disease Epidemiology, Imperial College London, London, UK; 2 Global Health Section, Department of Public Health, University of Copenhagen, Copenhagen, Denmark; 3 Centre for Medical Parasitology, Department of Immunology and Microbiology, University of Copenhagen, Copenhagen, Denmark; 4 Department of Infectious Disease, Copenhagen University Hospital, Copenhagen, Denmark; 5 Department of Biomedical Sciences, National Institute for Medical Research, Tanga, United Republic of Tanzania; 6 RTI International, Washington, District of Columbia, USA; 7 Malaria Elimination Initiative, Global Health Group, University of San FranciscO, San Francisco, California, USA; 8 Malaria Atlas Project, Oxford Big Data Institute, Li Ka Shing Centre for Health Information and Discovery, Nuffield Department of Medicine, University of Oxford, Oxford, UK; 9 Department of Pathogen Molecular Biology, London School of Hygiene and Tropical Medicine, London, UK

**Keywords:** malaria, epidemiology, parasitology, public health, systematic review

## Abstract

Artemether–lumefantrine (AL) and artesunate–amodiaquine (AS-AQ) are the most commonly used artemisinin-based combination therapies (ACT) for treatment of *Plasmodium falciparum* in Africa. Both treatments remain efficacious, but single nucleotide polymorphisms (SNPs) in the *Plasmodium falciparum* multidrug resistance 1 (*Pfmdr1*) gene may compromise sensitivity. AL and AS-AQ exert opposing selective pressures: parasites with genotype 86Y, Y184 and 1246Y are partially resistant to AS-AQ treatment, while N86, 184 F and D1246 are favoured by AL treatment. Through a systematic review, we identified 397 surveys measuring the prevalence of *Pfmdr1* polymorphisms at positions 86 184 or 1246 in 30 countries in Africa. Temporal trends in SNP frequencies after introduction of AL or AS-AQ as first-line treatment were analysed in 32 locations, and selection coefficients estimated. We examined associations between antimalarial policies, consumption, transmission intensity and rate of SNP selection. 1246Y frequency decreased on average more rapidly in locations where national policy recommended AL (median selection coefficient(*s*) of −0.083), compared with policies of AS-AQ or both AL and AS-AQ (median *s*=−0.035 and 0.021, p<0.001 respectively). 86Y frequency declined markedly after ACT policy introduction, with a borderline significant trend for a more rapid decline in countries with AL policies (p=0.055). However, these trends could also be explained by a difference in initial SNP frequencies at the time of ACT introduction. There were non-significant trends for faster selection of N86 and D1246 in areas with higher AL consumption and no trend with transmission intensity. Recorded consumption of AS-AQ was low in the locations and times *Pfmdr1* data were collected. SNP trends in countries with AL policies suggest a broad increase in sensitivity of parasites to AS-AQ, by 7–10 years after AL introduction. Observed rates of selection have implications for planning strategies to cycle drugs or use multiple first-line therapies to maintain drug efficacy.

**Key questions**

**What is already known?**

- The two antimalarial drugs most commonly used in sub-Saharan Africa, artemether–lumefantrine (AL) and artesunate–amodiaquine (AS-AQ), exhibit collateral sensitivity.
- Parasites with the YYY haplotype at positions 86, 184 and 1246 in the multidrug resistance transporter 1 (*Pfmdr1*) gene show reduced sensitivity to AS-AQ, while the NFD haplotype is associated with reduced sensitivity to AL.

**What are the new findings?**

- We find a widespread decline in the prevalence of the 86Y and 1246Y mutations, particularly in countries which recommended AL as first-line antimalarial treatment over the last ~10 years, suggesting slightly increased sensitivity to AS-AQ and slightly reduced sensitivity to AL.
- Different artemisinin-based combination therapies (ACT) first-line policies and historical frequencies of the mutations are significantly associated with rates of selection, but malaria transmission intensity is not.

**What do the new findings imply?**

- The observed rates of selection have implications for designing policies which exploit the collateral sensitivity of these two drugs, such as drug cycling, sequential ACT treatments or multiple first-line therapies.

## Introduction

For many years, the recommended first-line treatment for uncomplicated *Plasmodium falciparum* malaria was chloroquine but resistance emerged in South East Asia and South America in the 1950s, subsequently spreading through Africa in the 1970s–1980s.^1^ As a consequence, many African countries changed policy for treatment of uncomplicated *falciparum* malaria to recommend sulphadoxine–pyrimethamine (SP) instead. However, increasing clinical SP treatment failures in the late 1990s, particularly in East Africa, demanded a change in policy. Since 2001, WHO has recommended treating uncomplicated malaria with artemisinin-based combination therapies (ACTs), which are now adopted as policy in nearly all endemic countries.^2^ ACTs consist of a fast-acting artemisinin derivative that quickly reduces the parasite load, combined with a partner drug with a much longer half-life (eg, lumefantrine, amodiaquine, mefloquine, piperaquine) that will eliminate the remaining parasites and protect against the establishment of new infections for a period of time postprime infection.^3 4^ However, there is evidence of emerging artemisinin resistance in South-East Asia, shown by decreasing parasite clearance rates after treatment and also, increasingly, ACT treatment failure in areas where partner drug resistance has spread.^5 6^ Single nucleotide polymorphisms (SNPs) in the *P. falciparum* kelch 13 gene are associated with reduced susceptibility to artemisinin in vivo and in vitro.^7 8^ Although it has not yet been documented that such parasites occur in Africa, recent data from a few African countries show diminished efficacy of certain ACT combinations,^9 10^ thus, other *P. falciparum* genes may also be involved in ACT resistance.

Artemether–lumefantrine (AL) and artesunate–amodiaquine (AS-AQ) are by far the most commonly used ACTs in Africa,^2^ although access to these treatments still remains relatively low in many areas.^11^ Interestingly, these two drugs appear to exert opposing selection pressures on polymorphisms in the *P. falciparum* multidrug resistance transporter 1 (*Pfmdr1*) gene, which codes for transporter molecule P-glycoprotein homologue 1. Mutations in *Pfmdr1* were first shown to be important in chloroquine resistance^12^ and they have since been shown to have significant effects on tolerance and resistance to most other antimalarial drugs.^13–17^ In Africa, SNPs in codons N86Y, Y184F and D1246Y of *Pfmdr1* are most common and different haplotype combinations of these confer reduced sensitivity to different drugs. For instance, AQ alone and AS-AQ have been shown to select for *Pfmdr1* 86Y, Y184 and 1246Y (the YYY haplotype) while the AL combination selects for N86, 184F and D1246 (the NFD haplotype).^5 18–22^ Similar observations have been documented in in vitro and ex vivo susceptibility assays.^23–25^ A meta-analysis estimated that parasites with the N86 wildtype genotype are 4.7 times more likely to recrudesce after AL treatment than parasites with the 86Y genotype.^5^ In addition, early treatment failures that occur between day 2 and 3 post-treatment with AL and also another ACT, dihydroartemisinin (DHA)–piperaquine, were significantly associated with the *Pfmdr1* NFD haplotype in a study in Kenya.^26 27^ A recent study from Tanzania on reinfection post-treatment estimated that parasites harbouring the *Pfmdr1* NFD haplotype were able to withstand a 15-fold higher concentration of lumefantrine compared with parasites with the *Pfmdr1* YYY haplotype.^27^ The effect of the artemisinin component on selection is less clear. Gene editing suggests that *Pfmdr1* 86Y may increase parasite susceptibility to DHA, the active metabolite of artemisinin derivatives, although this only occurs in standard parasite growth inhibition experiments and not after the 6-hour DHA exposure assay which is more representative of exposure in vivo.^13^ *Pfmdr1* copy number variation is also associated with differential susceptibility to antimalarials, with multiple copies reducing sensitivity to lumefantrine and mefloquine. In Africa, multiple copies are rare in most surveys, with a few exceptions, notably a recent study in Ethiopia.^28^

The observed opposing selection pressure of AL and AS-AQ has led to interest in harnessing this effect to optimise malaria treatment efficacy. For instance, these two drugs could be cycled in national treatment policies,^29^ deployed simultaneously in populations^30^ or used sequentially in individual patients when the initial treatment fails.^31^ Planning for these strategies will benefit from understanding and quantifying the current direction and rates of change in these SNPs in populations in relation to ACT policies and consumption. Here, we collate and map all published *Pfmdr1* SNP data at positions 86, 184 and 1246 and analyse the effect of implementation of AS-AQ and AL on the temporal trends in the population prevalence of these SNPs across Africa.

## Methods

### Systematic review

A PubMed Search was carried out up to the 20 October 2017 using the following free text and MESH words: “Plasmodium falciparum”[Mesh] OR Plasmodium falciparum[Text Word]))) AND ((Malaria[Text Word] OR “Malaria”[Mesh] OR “Malaria, falciparum”[Mesh]))) AND (((single nucleotide polymorphisms[Text Word] OR “Polymorphism, Single Nucleotide”[Mesh] OR “*mdr* gene protein, Plasmodium” OR multidrug resistance gene* OR genetic variant* OR Plasmodium falciparum Polymorphisms[text word] OR Pfmdr*. The bibliographies of the articles were read to identify more relevant studies. We included studies published after 1990 which measured the prevalence of the *Pfmdr1* N86Y, Y184F or D1246Y in samples from African countries which reported the year of data collection. We excluded articles based on in vitro studies or case reports, or where samples had been collected post-treatment. When samples were collected over more than a year, and the data were not disaggregated, the year was set to the midpoint of the study years. Towards the end of our review, data from an independent review were made available on the Worldwide Antimalarial Resistance Network (WWARN) website.^32^ We cross-checked our data and used an additional variable available in the WWARN database: the proportion of samples containing mixed wild type and resistant parasites.

### Data on antimalarial policies and antimalarial consumption

Data on antimalarial drug policies by country and year were extracted from annual World Malaria Reports by the WHO^33^ back to 2008, and WHO country reports prior to this time.^34^ In some areas, there were delays between policy change and implementation,^35 36^ but we used the official date of policy introduction recorded by WHO for all countries for consistency. We categorised country first-line treatment policies at the time the *Pfmdr1* SNP data were collected as AL alone, AS-AQ alone or both AL and AS-AQ (the latter means that patients are given either AL or AS-AQ—from here on, we call this policy AL/AS-AQ). Countries were included in the analysis if they had had a consistent first-line ACT policy for at least 5 years and for longer than any other ACT policy.

Data on reported antimalarial drug consumption in children from 0 to 5 years old were obtained from Demographic and Health Surveys (DHS), Malaria Indicator Surveys (MIS)^37^ and Multiple Indicator Cluster surveys (MICS, Unicef) carried out from 1999 to the present.^38^ We summarised data at the national level, allowing for the weighted, stratified survey designs. We also used the estimated percent of febrile, rapid-diagnostic-test (RDT) positive 0–5-year-old children treated with ACT, by country and year that were recently released by the Malaria Atlas Project.^11^ These estimates take into account country-specific ACT procurement data as well as reported use during DHS, MIS and MICS surveys. For a further source of estimates of ACT consumption, we used data on the market share of different types of antimalarials measured by ACTWatch in national surveys, where ‘% market share’ is defined as “% adult equivalent treatment dosages of a given type of antimalarial, out of the total sold or distributed in the previous week’.^39^ ACTWatch surveyed a representative sample of both public and private outlets providing or selling antimalarials. Some surveys recorded the use of ACT but did not specify a type. Here, we assumed that the ACT taken was the first-line antimalarial in the country policy at the time if their policy contained only a single ACT and excluded these data if the country policy recommended more than one first-line ACT.

### Statistical analysis

*Pfmdr1* mutation prevalence data were geolocated and mapped using the maptools package in R software V.3.4.0.^40^ We analysed the rate and direction of selection before and after changing to ACT policy for each country and location, including locations which had two or more measures of a mutation (at least one non-zero) at different time points spanning at least 3 years during either the pre-ACT or post-ACT policy time periods. We grouped locations that were within 50 km of each other, with the exception of western Kenya, where some measures 100 km apart were aggregated in the published data, and therefore all data from this region were grouped. For these time series data, we extracted not only the prevalence of the mutations in infected individuals but also the frequencies of mutations in the parasite population reported by the original studies where available. For example, some simply exclude mixed wild type-resistant infections, or assume that these infections contain one wild type and one resistant parasite clone, or use statistical methods to estimate frequencies. Otherwise, frequencies of mutations were estimated where possible using a previously published method.^41^ This method requires an estimate of local slide prevalence in order to estimate the distribution of the number of parasite clones per infected person. We used the Malaria Atlas Project estimates of slide prevalence in the year and location that each study was done, within a 20 km radius of the study location.^42^ Frequency estimation using this previously published method has been found to be robust as long as the proportion of mixed wild type-resistant infections is known,^41^ and therefore, we did not estimate frequencies for studies not reporting these data.

Selection coefficients (the % change in relative frequency of the mutant genotype per parasite generation) were calculated for each mutation at each location, by linear regression of the log ratio of mutant to wild type frequency over time.^43 44^ We assumed three parasite generations per year.^45^ The regression was weighted by the inverse variance of the log frequency ratio from each survey, to allow for different sample sizes. We also computed selection coefficients using prevalence of mutation data in individuals instead of frequency in parasites, for comparison and found this made very little difference in the large majority of locations (n=20 locations with both frequency and prevalence data, online supplementary information). Therefore, in locations where frequency data were not available, we used prevalence data instead to compute selection coefficients (n=12 locations). We allowed selection coefficients to be different during pre-ACT versus post-ACT periods but otherwise assumed constant selection over time in each location. To test for a difference in mean selection coefficients according to different potential drivers of selection, we used a multilevel model with random intercepts and slopes for each location^46 47^ and tested for a difference in slopes in areas with different covariate categories using interaction terms. Due to small numbers of countries with AS-AQ policies, we grouped these together with countries using both AL/AS-AQ for some analyses. Other covariates tested were the initial frequency/prevalence of the SNP at the start of the time series, antimalarial use (ACT use estimated by the Malaria Atlas Project, chloroquine use estimates from DHS, MIS and MICS surveys) and transmission intensity (measured by slide prevalence in 2–10 year olds.^42^ Antimalarial use and slide prevalence for each location were computed as the mean of all data points/estimates measured during the time period where a SNP time series was available, and for antimalarial use, we also included any measures within 2 years of the start and end of the time period, because there were often several years between surveys. The only exception was chloroquine use in Rwanda, which was most recently measured as zero in 2005. We also assumed zero use during later years, since it is unlikely that chloroquine use would have increased after 2005.

10.1136/bmjgh-2018-000999.supp1 Supplementary data

## Results

### Systematic review results

**Figure 1:**
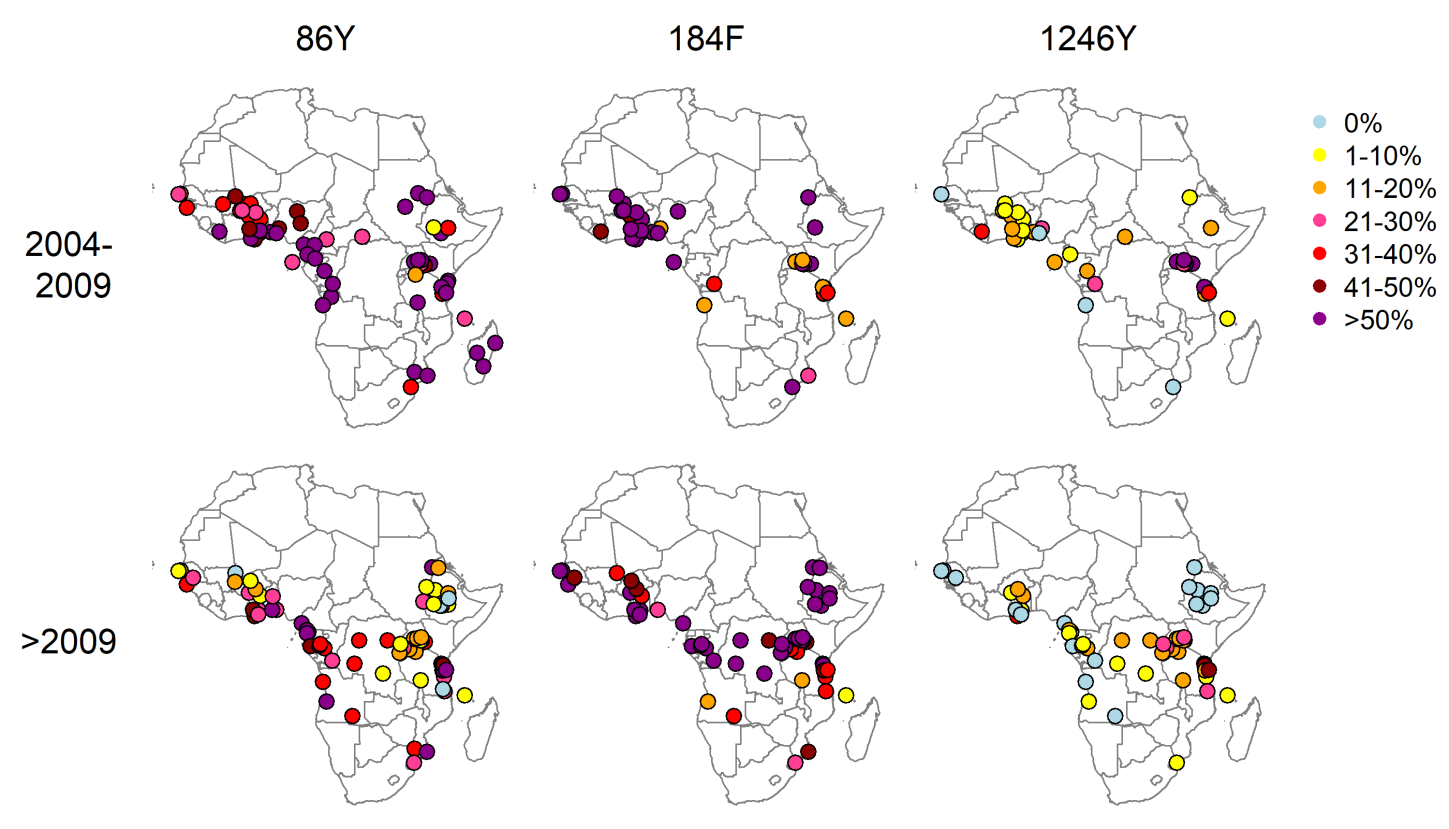
Prevalence of the 86Y, 1246Y and 184F *Plasmodium falciparum* multidrug resistance 1 (*Pfmdr1*) mutants in infected individuals in Africa over different time periods. Prevalences before 2004 are shown in online supplementary figure S1.

**Figure 2:**
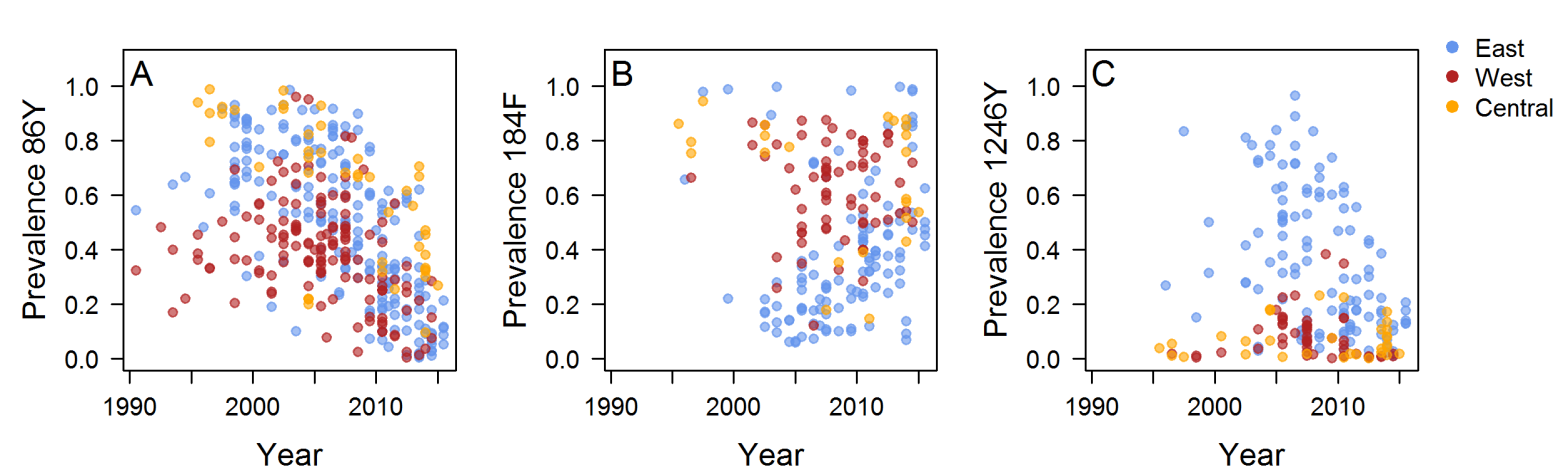
The frequency or prevalence of *Plasmodium falciparum* multidrug resistance 1 (*Pfmdr1*) mutations over time. Where possible, mutant frequencies were extracted from publications or estimated (see the Methods section), otherwise mutant prevalence data are shown. Locations are coloured by geographical region and include the following countries. East Africa: Uganda, Tanzania, Zanzibar, Tanzania, mainland, Swaziland, Sudan, Rwanda, Mozambique, Malawi, Madagascar, Kenya, Ethiopia, Eritrea, Comoros; West Africa: Senegal, Nigeria, Mauritania, Mali, Liberia, Guinea-Bissau, Ghana, Gambia, Burkina Faso, Benin; Central Africa: Sao Tome and Principe, Republic of Congo, Gabon, Equatorial Guinea, Democratic Republic of Congo, Central African Republic, Cameroon, Angola. See also online supplementary figure S2-4 for the data separately by location and online supplementary data for the full data.

The literature search identified 930 articles, of which 171 met the inclusion criteria. Data were extracted from 397 surveys measuring the prevalence or frequency of at least one *Pfmdr1* polymorphism in 30 countries published up to October 2017. There were 397 214 and 211 surveys measuring the 86Y, 184F and 1246Y mutations, respectively, and we obtained mutation frequency estimates for 229, 129 and 146 surveys (figure 1, online supplementary figure 1, online supplementary data). The number and time points of surveys varied greatly between locations, with sparser coverage in parts of central Africa. The 86Y and 184F mutations are ubiquitous, but higher levels of 86Y were more often recorded in East and Central Africa compared with West Africa during the 1990s and early 2000s, whereas West Africa tended to have higher levels of 184F (figure 2A,B). The 1246Y mutation occurred on average at lower levels than the other two *Pfmdr1* polymorphisms and was not detected at all in 17% of surveys. The 1246Y is found in all regions of the continent, but higher prevalences of the mutation were detected mainly in East Africa (figure 2C), with a prevalence of 1246Y of over 50% only being detected in Kenya, Uganda and Tanzania.

10.1136/bmjgh-2018-000999.supp2 Supplementary data

### Quantifying SNP selection over time by ACT policy

**Figure 3:**
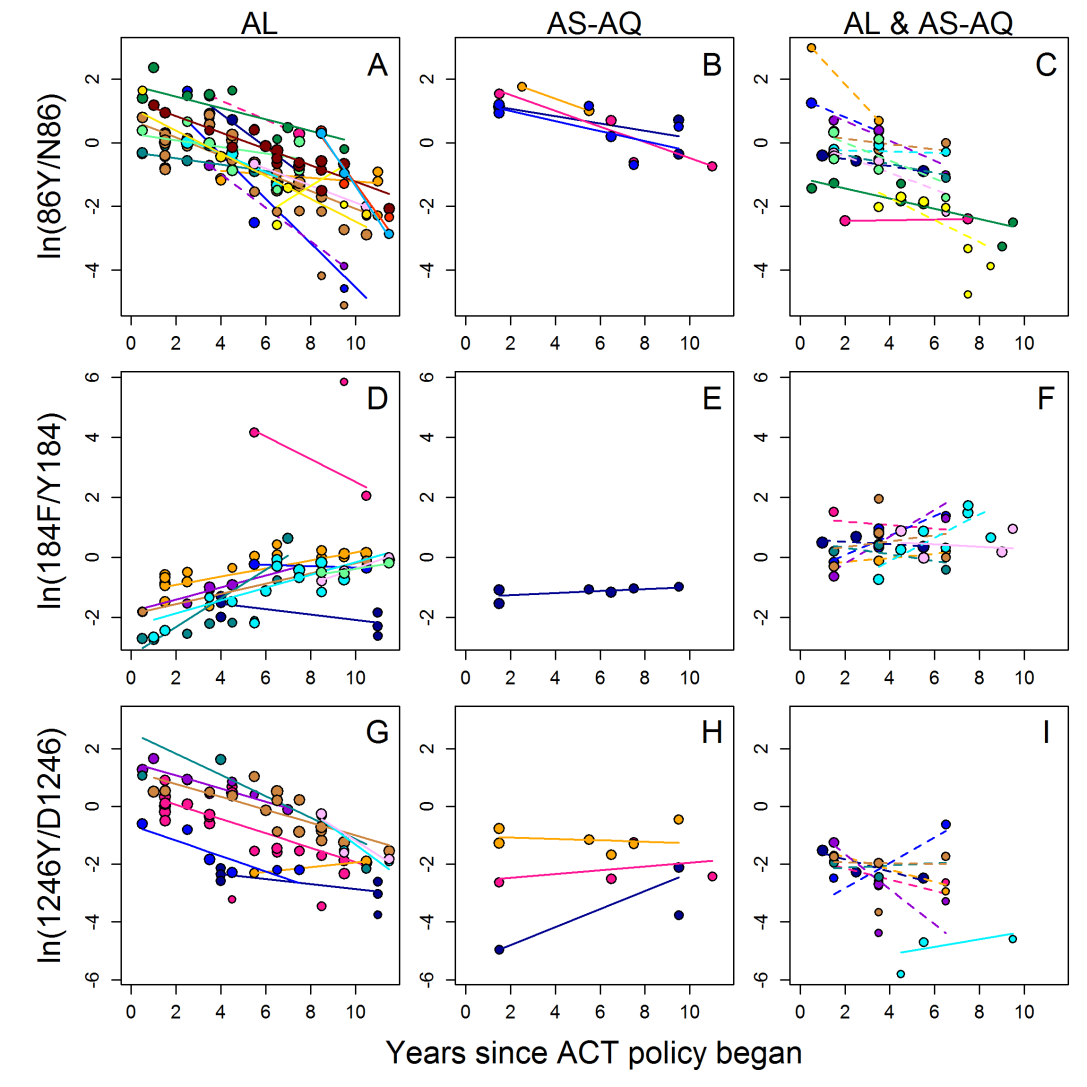
Selection of *Plasmodium falciparum* multidrug resistance 1 (*Pfmdr1*) polymorphisms in 32 locations within countries with different first-line artemisinin-based combination therapies (ACT) policies, as shown by the change in the ratio of *Pfmdr1* mutant to wild type frequency/prevalence over time since introduction of ACT policy. Panels show natural log mutant:wild type ratios at *Pfmdr1* positions 86 (A–C), 184 (D–F) and 1246 (G–I). Areas had (A,D,G): artemether–lumefantrine (AL) policy, (B,E,H) artesunate–amodiaquine (AS-AQ) policy or (C,F,I) both AL and AS-AQ policy. Circles indicate the data, coloured by location and lines the fitted weighted linear regression at each location (see online supplementary figure S5-7 for the data separately by location). The slope on the natural log scale indicates the selection coefficient at each location. Where available, frequencies of each allele were used to estimate the slopes (solid lines), or otherwise prevalence data were used (dashed lines – see also the Methods section).

Figure 3 shows data on the three *Pfmdr1* mutations over time in locations which had multiple measures over at least 3 years, after introduction of ACT policy (n=177 surveys). Additionally, online supplementary figure S2-7 show each location separately. Each location was categorised according to whether the country had a first-line policy of treating all patients with AL, all with AS-AQ, or patients with either AL or AS-AQ (the latter policy is referred to as AL/AS-AQ) (figure 3, online supplementary figure S3 and 8). The full data across the continent suggest a marked decline in the frequency/prevalence of the 86Y mutant after about 2005 (figure 2A), and this was confirmed in 32 locations (15 countries) with longitudinal data after ACT introduction, where 30 of the 32 locations observed a decline in 86Y (figure 3A–C). The decline was on average fastest in locations within countries which had a policy of AL as first line treatment (n=16), with a median selection coefficient of −0.096 (figure 3A–C). However, the decline was also observed in locations within countries with AS-AQ (n=4) or AL/AS-AQ policies (n=12), with median selection coefficients of −0.068 and −0.070, respectively (borderline statistically significant difference from AL countries, p=0.055, online supplementary table S1). However, higher initial 86Y frequencies at the start of the observed time period were significantly associated with a more rapid decline over time (p<0.001) (figure 2, online supplementary figure S2 and 9 & table S1) and were on average different in countries with different ACT policies. After adjusting for this variable, countries with AL policy still had more rapid declines in 86Y frequency, but the difference was reduced and was not statistically significant (p=0.32).

By contrast, the 184F mutation increased in frequency/prevalence in 13 out of 20 locations with available data: these included countries in East Africa as well as parts of Ghana and Senegal (figure 3, online supplementary figure S6). In countries with AL as first-line policy, the median selection coefficient for the mutant was highest at 0.051 per generation, while in countries with AS-AQ (data only available from Tanzania, Zanzibar for this mutant) or AL and AS-AQ policy, the median selection coefficients were 0.011 and 0.023, respectively. This difference between selection coefficients in countries recommending AL versus AS-AQ or AL/AS-AQ was not statistically significant (p=0.280, online supplementary table S1).

1246Y mutant frequency/prevalence decreased in most locations within countries with AL policies (8 out of 9) (figures 2C and 3G) but showed more variable patterns in areas recommending AL/AS-AQ or AS-AQ alone (figures 2C, 3H and I). The most rapid selection of the wild type D1246 occurred mainly in East African countries (Kenya, Tanzania, Uganda) but also in parts of Ghana (online supplementary figure S4). The median selection coefficient of 1246Y in areas with AL policy was −0.083 (n=9), but in areas recommending AL/AS-AQ or AS-AQ alone (n=8 and 3, respectively), the trends were on average more stable, with median selection coefficients of −0.035 and 0.021, respectively (a statistically significant difference between selection coefficients in AL versus AS-AQ or AL/AS-AQ countries, p<0.001, online supplementary table S1). However, again the difference could be explained by different initial frequencies as well as by different drug policies. Mutant 1246Y was significantly higher at the time of ACT introduction in locations within countries which chose AL compared with the other policy options (p=0.004 figure 3G–I), and its initial frequency was also significantly negatively associated with the subsequent selection (p<0.001 online supplementary figure S9). After adjusting for starting frequency, there was no significant effect of drug policy on selection coefficients (p=0.66).

### SNP selection before introducing ACT policy

Before ACTs were introduced as first-line policy, trends in the 86Y mutation were variable but remained at relatively stable levels in most locations with available data (n=16, median selection coefficient −0.006, online supplementary figure S10). Few locations had available data on 184F and 1246Y frequencies before ACT introduction, precluding further analysis (online supplementary figure S11 and S12).

### Quantifying SNP selection over time by ACT and chloroquine consumption and transmission intensity

**Figure 4:**
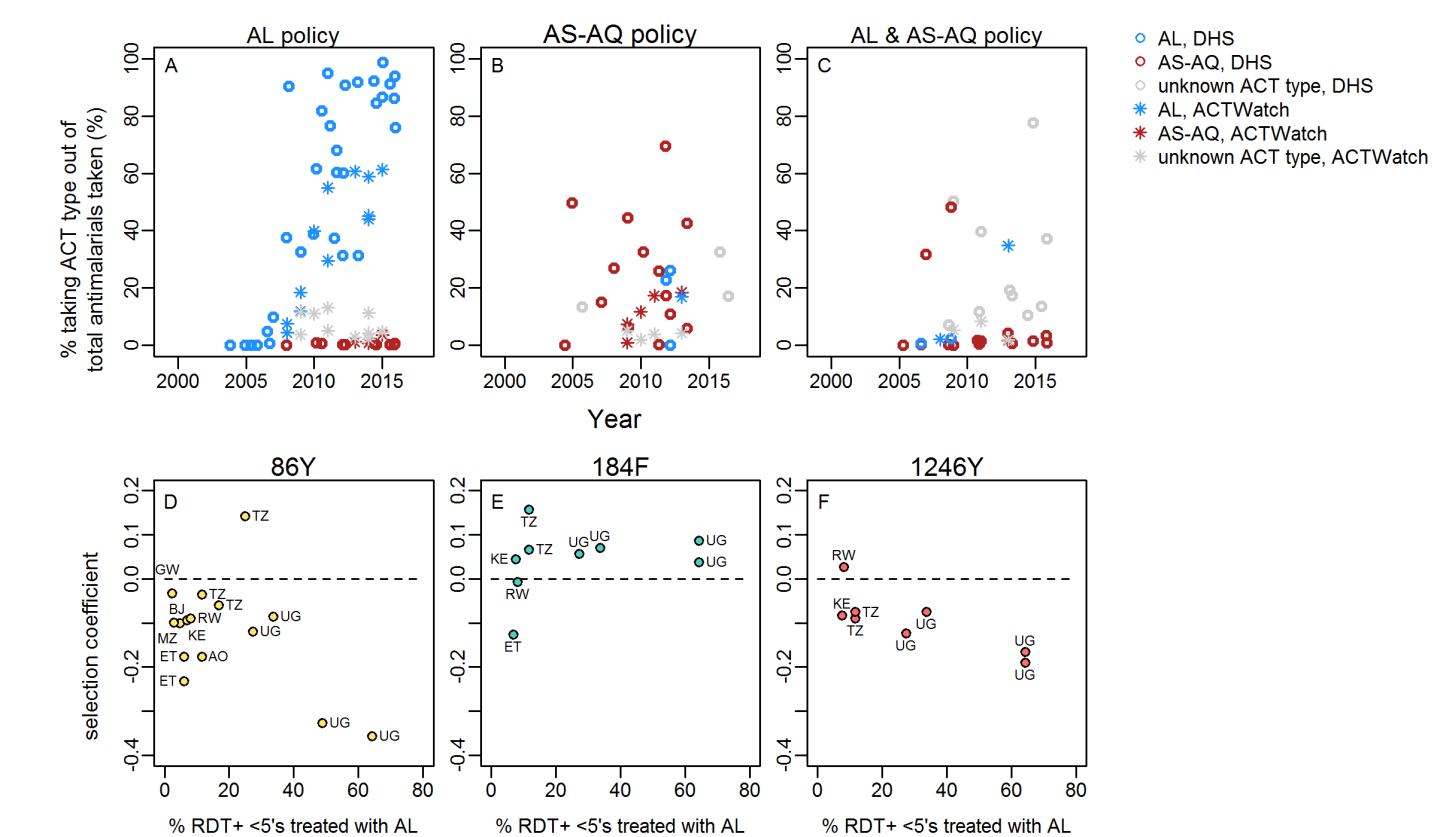
Consumption of artemisinin-based combination therapies (ACT) and *Plasmodium falciparum* multidrug resistance 1 (*Pfmdr1*) single nucleotide polymorphism (SNP) selection. Panels A–C: The proportion of all antimalarials taken/given which are artemether–lumefantrine (AL) (blue), artesunate–amodiaquine (AS-AQ) (red) or ACT of unknown type (grey, may include AL, AS-AQ or other), according to national first-line policy. Data are either self-reported antimalarial use by caregivers of children aged 0–5 years recorded in national household surveys (DHS) or market share of ACT type out of all antimalarials sold/prescribed as recorded by representative national surveys of public and private dispensing outlets (ACTWatch). Data are shown for all countries with any *Pfmdr1* data in our review. Legend indicates colours in panels A–C. Panels D–F: estimated AL consumption and selection of *Pfmdr1* SNPs in countries with AL first-line policies. Selection coefficients for each SNP were estimated in locations with at least two measures of SNP frequency/prevalence over time periods spanning at least 3 years. The dashed line indicates zero change in frequency. AL consumption is the mean estimated % febrile RDT-positive 0–5 year olds taking ACT during the time period over which SNP trends were available for each location, from analysis by Bennett *et al*,^11^ assuming that all ACTs taken in these countries are the first-line policy, AL. None of the associations between AL consumption and SNP selection are statistically significant. Countries are indicated: AO: Angola, BJ: Benin, ET: Ethiopia, KE: Kenya, MZ: Mozambique, RW: Rwanda, TZ: Tanzania, UG: Uganda.

DHS and MIS surveys show the changing types of antimalarial drugs used over time in 0–5 year olds, with a clear scale up in access to ACTs in many countries. Figure 4 shows ACT consumption data from countries with available *Pfmdr1* SNP data and different ACT first-line policies. The change in ACT consumption occurred at variable rates in different countries. A noticeable trend was the generally low use of AS-AQ relative to AL, even in countries which recommend this drug in their first-line policy. A further trend was generally much higher estimated access to ACTs in countries with AL first-line policies (figure 4). Moreover, in countries with available data on *Pfmdr1* trends over time, the average mean % of RDT+fever cases which received ACT (during the time period the *Pfmdr1* data were gathered; estimates from the Malaria Atlas Project^11^) was 19% in countries recommending AL alone but only 3% in countries recommending AS-AQ or AL/AS-AQ.

In countries with AL policy, there was a slight trend that locations with higher estimated AL coverage among cases^11^ (>20%) during the time period when *Pfmdr1* SNP trends were measured showed more negative selection of 1246Y although this was not statistically significant(figure 4, online supplementary table S1). Selection of 86Y and 184F were not associated with AL coverage. There were not enough data from countries with AS-AQ or AL/AS-AQ policies to analyse associations of ACT consumption with selection.

Levels of chloroquine consumption among under 5 year olds (% taking chloroquine out of all those taking antimalarials) during the post-ACT period^37^ were lower in the countries with AL policies included in our analysis with a mean of 14% vs 28% in countries with AS-AQ or AL/AS-AQ policies. There was a slight trend for higher chloroquine consumption in areas with lower initial 1246Y frequencies at the start of the post-ACT data (p=0.048). However, chloroquine consumption was not associated with rate of selection of any of the SNPs, either in univariate or multivariate analysis (online supplementary table S1). Transmission intensity (estimated slide prevalence among 2–10 year olds,^42^ was lower on average in locations within countries recommending AL at 16% slide prevalence, compared with 40% in locations with AS-AQ or AL/AS-AQ policies. Transmission intensity was not associated with rate of selection of any SNPs in univariate or multivariate models (online supplementary table S1).

### Haplotypes and correlations between SNPs

**Figure 5:**
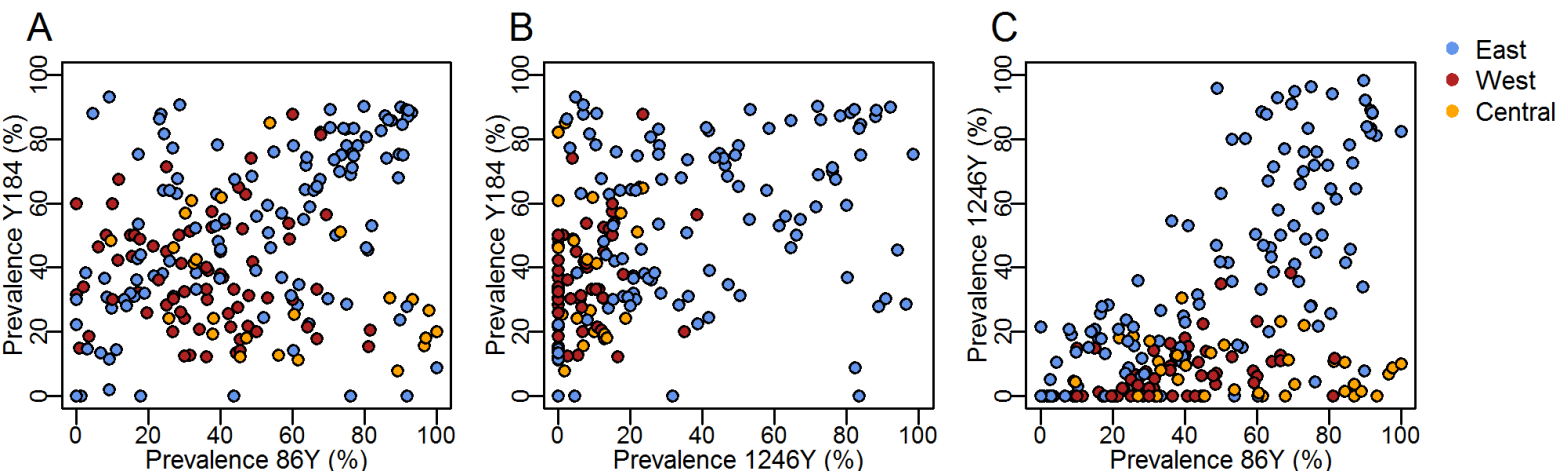
Relationship between prevalence of different *Plasmodium falciparum* multidrug resistance 1 (*Pfmdr1*) single nucleotide polymorphisms (86Y, Y184, 1246Y), measured in the same surveys and coloured by geographical region as in figure 2. Here, we show the wild type Y184 prevalence and its relationship with 86Y and 1246Y mutant prevalence in the interests of highlighting possible occurrence of the *Pfmdr1* YYY haplotype by region (exact haplotype frequencies are not known in the majority of surveys due to mixed infections).

To complement the analysis of individual SNPs and understand how linkage between *Pfmdr1* polymorphisms may contribute to selection, we examined correlations of the prevalence of the three SNPs in East, West and Central Africa as well as haplotype frequencies in a subset of locations with available time series. 184F prevalence was negatively correlated with 1246Y (p<0.001) (figure 5B). The 86Y and 1246Y mutation prevalences show a strong positive correlation (p<0.001) whereby 1246Y was only present at higher levels when 86Y was also present at higher prevalence, which occurred predominantly in East Africa (figure 5C).

A total of 12 locations in eight countries reported multiple measures of haplotypes over time (online supplementary figure S13). Five countries with haplotype data series used AL as the first-line treatment (Comoros, Swaziland, Kenya, Tanzania and Uganda). The general trend in these locations was a decrease in at least one of the YYY, YFY and YYD haplotypes and a rise in the NFD and NYD haplotypes. A different pattern was seen in the Comoros, where the YFY haplotype initially dominated in 2006, followed by a rise in the YFD and YYD haplotypes and eventually higher levels of NYD in 2014. Two locations with available haplotype data, Zanzibar (Tanzania) and Madagascar, used AS-AQ as a first-line treatment. In both, there was an unexpected increase of the NYD and NFD haplotypes after ACT introduction.

## Discussion

During the era of chloroquine use against malaria, the 86Y and 1246Y mutations in the *Pfmdr1* gene were selected^12^, and the 86Y became widespread in Africa. After the change in drug policy from chloroquine to ACT, we find that the frequency of 86Y declined markedly over the next 10 years in most locations with available data. 1246Y has declined in countries using AL, while the 184F mutation increased in frequency in many areas. Our analysis indicates that countries with first-line AL policies showed significantly more rapid decline of 1246Y, and borderline significant more rapid decline in 86Y, compared with countries recommending AS-AQ or AL/AS-AQ as first-line policy. Furthermore, within countries recommending AL, there was a non-significant trend towards more rapid decline of the 1246Y in countries with higher estimated consumption of AL per malaria case (>20% coverage). The trends suggest a slight increase in susceptibility of parasites to AS-AQ in countries which have implemented AL alone, consistent with trial results.^29^ It is unclear whether the different rates of selection of SNPs observed in countries recommending AS-AQ or AL/AS-AQ were related to AS-AQ itself. An alternative explanation was the very low estimated use of ACTs in countries with AS-AQ or AL/AS-AQ policies (only 3% of cases receiving ACT on average during the time periods examined, and a low average recorded use of AS-AQ in household surveys).^37^ Also, the selection pressure exerted by AS-AQ may not be simply in one direction, since although amodiaquine is thought to select for the 86Y and 1246Y, there is some in vitro evidence suggesting that the artemisinin component selects for the wild type genotypes at these positions.^48^

We identified several other differences in countries which recommend AL compared with countries recommending AS-AQ or AL/AS-AQ, which may have also driven SNP trends. It is likely that the original choice of AL versus AS-AQ for national policy in many countries was influenced by their levels of amodiaquine and chloroquine resistance at the time. In particular, countries with AS-AQ as a component of their first-line treatment policy were significantly more likely to have lower frequencies of the 1246Y mutation at the time of ACT introduction. This factor also explains some of the difference in SNP trends in countries recommending AS-AQ or AL/AS-AQ, that is, there was simply less potential for 1246Y to decline because initial frequencies were so low. Initial SNP frequencies were associated with subsequent rates of change in frequency for all three mutations, which could be due to regression to the mean (locations with initial outlier measurements later have more precise measures closer to the true value), or due to epidemiological reasons, for example, variations in treatment access between populations. That is, countries with high use of chloroquine could have selected 86Y and 1246Y to high levels, followed by subsequent similarly high use of AL leading to a more rapid decline in those SNPs.

The widespread withdrawal of chloroquine across Africa is likely to have caused a decline in 86Y and 1246Y frequencies independently of ACT introduction, given possible fitness costs of these mutations.^49–51^ This effect may explain why the decline was seen even in countries recommending AS-AQ. We examined whether countries which withdrew chloroquine more quickly after officially switching to ACT might show more rapid rates of decline in these mutations, but we did not find quantitative evidence for this in our analysis. However, there are limitations in the estimates of chloroquine consumption (see below). Countries with AS-AQ or AL/AS-AQ policies included in our analysis had on average higher chloroquine consumption, probably because they had lower initial prevalence of 1246Y, and thus better chloroquine efficacy.

We focused on separate SNPs in our analysis, due to paucity of data on haplotypes, although there is genetic linkage between these loci. Our review data suggest that the 1246Y has only spread to high levels where the 86Y was highly prevalent. SNPs at position 86 and 184 have previously been shown to be co-selected,^52^ although here only 86Y selection showed an association with ACT policy. Although the limited available haplotype data generally confirmed the SNP trends, showing an increase in NFD haplotypes in AL countries, the underlying dynamics may be more complex (eg, as shown for selection after DHA–piperaquine^53^) and depend on the past frequencies of haplotypes as well as importation of different haplotypes. Future analysis for example, of microsatellite data will elucidate genetic lineages of *Pfmdr1* haplotypes, while gene editing and phenotype testing could clarify whether each of the three loci confer changes in drug sensitivity, or rather fitness, or are simply co-selected. In addition, sensitivity to AL and AS-AQ is associated with mutations in the *P. falciparum* chloroquine resistance transporte (*Pfcrt*) gene and *Pfmdr1* copy number variation, which we did not consider here.^5^ Further analysis is required to define how mutations in these two genes are co-selected over time. Not all research laboratories are equipped to measure copy number variation which may mean that multiple copies are under-reported in Africa.

Influx of parasites from neighbouring countries is likely to influence SNP trends, particularly in lower transmission areas where a higher proportion of parasites are imported. AL countries are predominantly in East Africa, AS-AQ countries in central Africa and AL/AS-AQ countries in West Africa, so that it is difficult to distinguish the roles of spatial spread and drug pressure. Morris *et al* 54 and Froberg *et al* 48 studied the potential effect of spatial mixing in Zanzibar, Tanzania, where AS-AQ is used as first-line treatment, in contrast to mainland Tanzania where AL is first -line. Their studies showed reductions in prevalence of markers associated with amodiaquine resistance in Zanzibar, despite the use of AS-AQ as first-line treatment for many years (data also included in our analysis). Continued use of AL as second line, and its potential availability in the private sector could have contributed to these trends, but Zanzibar’s current low malaria prevalence^55^ and frequent travel to and from the mainland suggest a potential role for spatial spread of parasites. Spatial spread of parasites may also explain why we did not observe any clear differences in rate of selection by transmission intensity setting in the *Pfmdr1* SNP trends either in univariate analysis or after adjusting for drug policy or drug consumption. Differences are expected based on theoretical^56–58^ and some empirical results,^57^ but if parasites rapidly diffuse across a large enough area, differences between transmission settings might be quickly evened out.^59^

The data and analysis used here have limitations which may explain the absence of some expected associations, such as have been seen in multicountry standardised studies of antibiotic resistance and antibiotic consumption.^60^ The data on drug consumption from household surveys are an excellent resource and give access to standardised measures across the continent. However, drug consumption is self-reported, which may reduce accuracy and surveys are relatively infrequent in some locations. Furthermore, these measures were from under 5 year olds, and adults often tend to use different, cheaper drugs like SP.^39^ We used time since ACT policy introduction as a variable in our analysis, but the speed and coverage at which ACT policies are implemented varied in different countries.^36^ The presence of substandard or falsified ACTs in some countries may also affect SNP trends, but there are not sufficiently detailed data over time and in enough countries to include this factor in our analysis.^61^

A large number of *Pfmdr1* surveys have been conducted, but these are not necessarily representative of the continent since they tend to be concentrated in particular countries. The measures are also not standardised, including study populations of different ages and source populations (clinical/community), which may have different mutation prevalence.^62^ We made the simplistic assumption of constant selection of SNPs after introduction of ACT policy, given the lack of continuous measures of drug consumption and resistance, but could fit most longitudinal *Pfmdr1* data series reasonably well. We assumed a constant of three parasite generations per year. Generation time may vary by study setting, for example areas with higher overall access to antimalarial treatment may have on average shorter infections and generation times.^63^ This parameter is not well characterised but may affect our comparison of areas.

Many rich longitudinal datasets in different countries contributed to the trends analysed here. The most detailed data were often from countries with AL policy such as Kenya, Tanzania and Mozambique. The original publications, as well as noting overall increases in the N86 and often the 184F and D1246 when AL was introduced, sometimes reported detailed local information such as fluctuations in drug availability, that we did not capture since it was not available for most included locations. In Kenya, Angola and Guinea-Bissau after AL policy introduction, amodiaquine, SP–amodiaquine or quinine were reportedly used in some areas during periods when AL was not available, which the authors suggest may have slowed selection of the NFD haplotype.^35 36 64–67^ Senegal, which had a policy of AL/AS-AQ reported that sometimes only one ACT was in use due to stock-outs, and that when both were available, AL was used more often because it was better tolerated.^68 69^ Longitudinal studies in countries using AS-AQ noted more stable trends in *Pfmdr1* SNP prevalence, which was attributed to ongoing use of alternative antimalarials, selection of wild type N86 and D1246 by the artemisinin component and importation of parasites.^48 70 71^

The trends in *Pfmdr1* SNPs at a population level in Africa can help to identify how opposing selection pressures exerted by these two ACT combinations may be harnessed by drug policies to improve treatment outcomes. Drug cycling is one proposed option, for example countries which have used AL and reduced the frequency of the YYY haplotype may consider switching to AS-AQ.^29^ Several countries already recommend both AL and AS-AQ as first-line therapy (online supplementary figure S8), although the optimal proportion of each drug provided is unclear, and might depend on relative selection strength of each drug and the levels of treatment failure after different drug–genotype combinations.^72^ A strategy of retreating treatment failures with the alternative combination (AL failures with AS-AQ, and vice versa) has been proposed.^31^ In the future, enhanced diagnostics are likely to optimise treatment choice for each patient dependent on the genotype of their infection.^73^ Clinical trials are also studying the efficacy of sequential treatment with two different ACTs,^74^ and triple combination therapy containing an artemisinin derivative, lumefantrine and amodiaquine.^75^ Given the importance of partner drug effectiveness in preventing ACT treatment failure as well as artemisinin resistance spread, it will be important to continue and expand monitoring of both resistance as well as rates of different ACT consumption.
