# Supplementary material for: Emerging implications of policies on malaria treatment: genetic changes in the Pfmdr-1 gene affecting susceptibility to artemether–lumefantrine and artesunate–amodiaquine in Africa
Source: BMJ Glob Health. 2018 Oct 19;3(5):e000999. doi: 10.1136/bmjgh-2018-000999 (PMC6202998; doi:10.1136/bmjgh-2018-000999)
Supplement: Supplementary data [file bmjgh-2018-000999supp001.pdf]

Supplementary Figures & Table

Emerging implications of policies on malaria treatment: genetic changes in the *Pfmdr-1* gene affecting susceptibility to artemether-lumefantrine and artesunate-amodiaquine in Africa

Lucy Okell[1*] Lisa Malene Reiter[2*], Lene Sandø Ebbe[3], Vito Baraka[4], Donal Bisanzio[5], Oliver Watson[1], Adam Bennett[6], Robert Verity[1], Peter Gething[7], Cally Roper[8], Michael Alifrangis[3]

Institutions:

[1] MRC Centre for Outbreak Analysis and Modelling, Department of Infectious Disease Epidemiology, Imperial College London

[2]Global Health Section, Department of Public Health, University of Copenhagen, Copenhagen, Denmark

[3]Centre for Medical Parasitology, Department of Immunology and Microbiology, University of Copenhagen, Denmark; Department of Infectious Disease, Copenhagen University Hospital, Denmark

[4]National Institute for Medical Research, Tanga Centre, P. O. Box 5004, Tanga, United Republic of Tanzania

[5]RTI International, Washington, DC, USA

[6] Malaria Elimination Initiative, Global Health Group, University of San Francisco, San Francisco, CA, USA

[7] Oxford Big Data Institute, Li Ka Shing Centre for Health Information and Discovery, Nuffield Department of Medicine, University of Oxford, Oxford, UK

[8] London School of Hygiene and Tropical Medicine

* These authors contributed equally

Figure S1. Prevalence of the 86Y, 1246Y and 184F *Pfmdr1* mutants in infected individuals in Africa before 2004. Prevalences after 2004 are shown in the main text Figure 1.

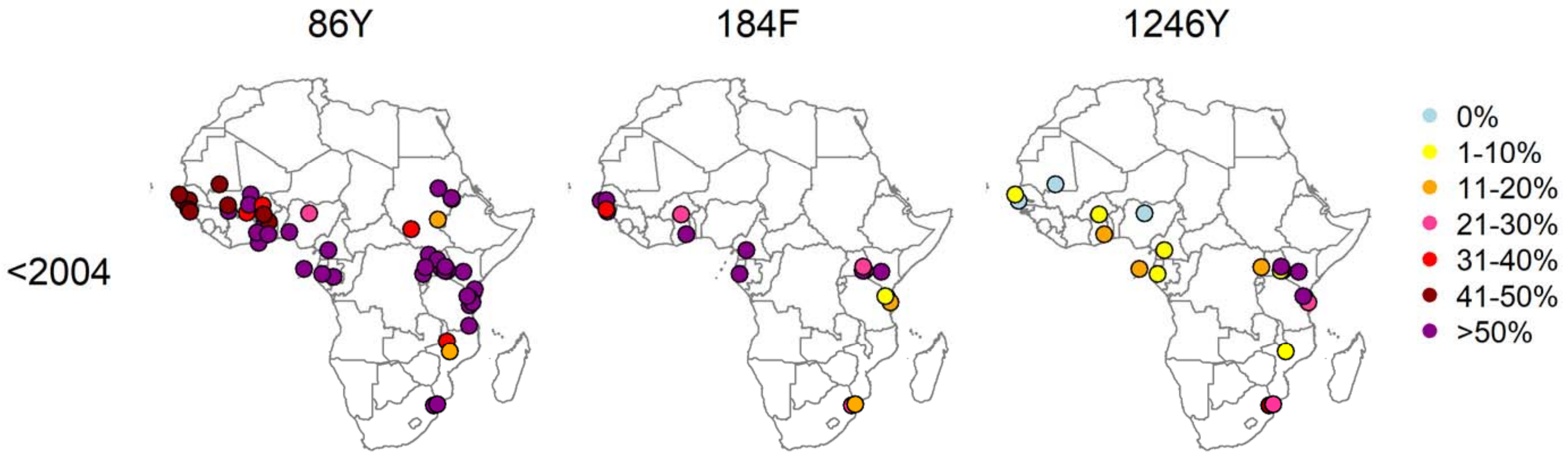

Figure S2. 86Y mutant frequency (where available – see Methods) and prevalence after introduction of ACT policy in locations which measured mutant prevalence at least twice, at least 3 years apart after the introduction of ACT policy, and with at least one non-zero observation. Panel outline colours indicate which ACT was first-line. Burkina Faso BD = Bobo-Dioulasso.

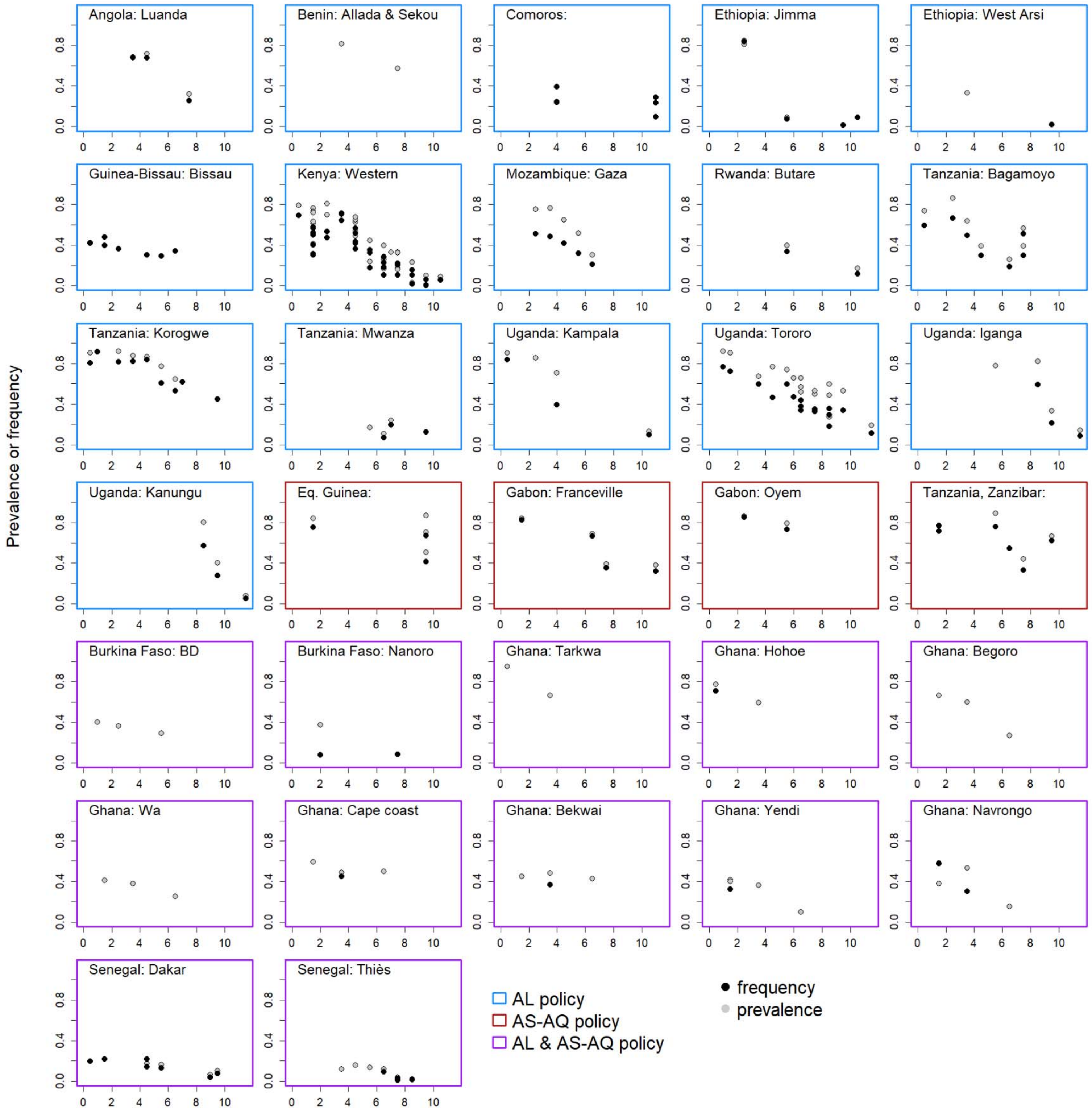

Figure S3. 184F mutant frequency (where available – see Methods) and prevalence after introduction of ACT policy in locations which measured mutant prevalence at least twice, at least 3 years apart after the introduction of ACT policy, and with at least one non-zero observation. Panel outline colours indicate which ACT was first-line.

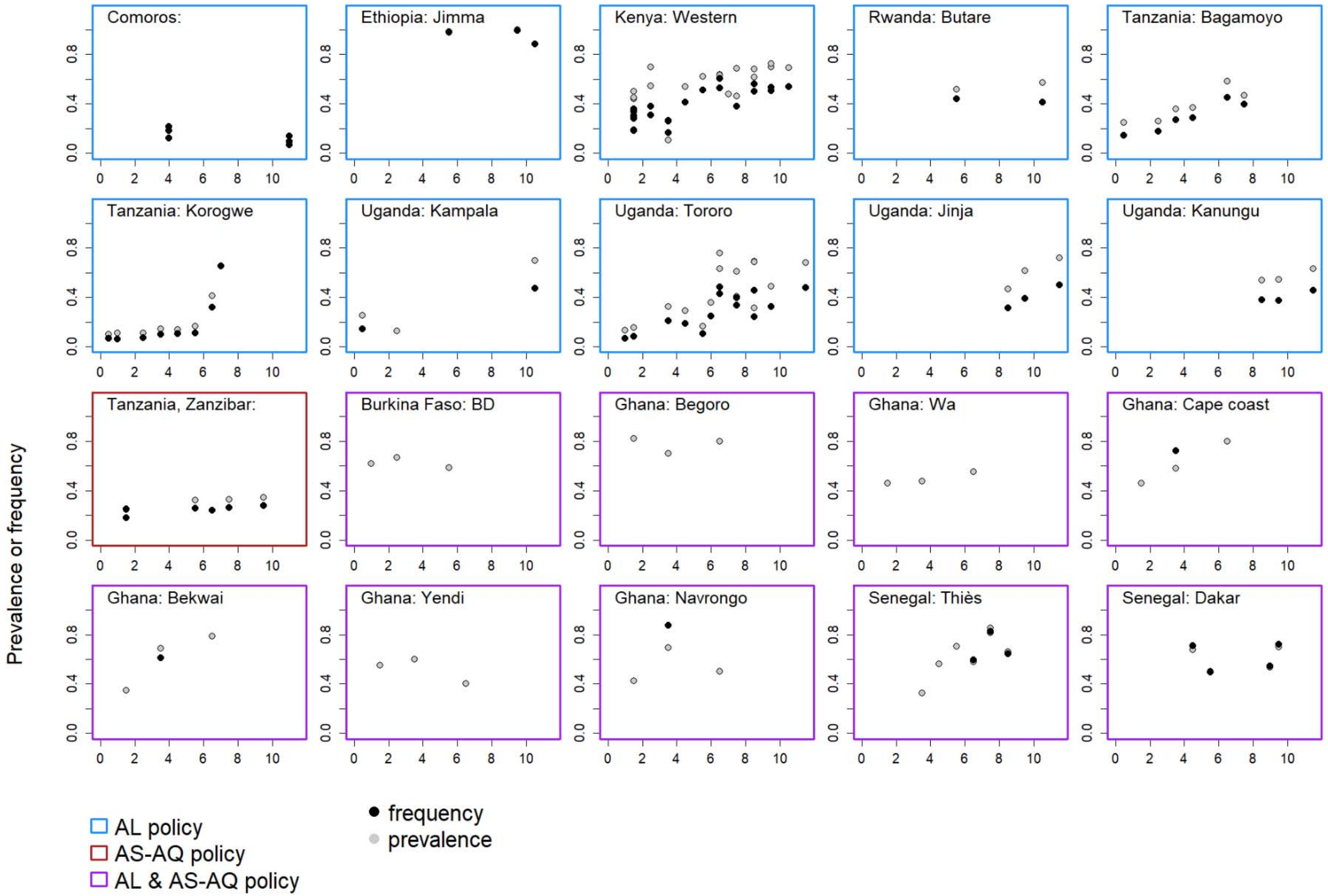

Figure S4. 1246Y mutant frequency (where available – see Methods) and prevalence after introduction of ACT policy in locations which measured mutant prevalence at least twice, at least 3 years apart after the introduction of ACT policy, and with at least one non-zero observation. Panel outline colours indicate which ACT was first-line.

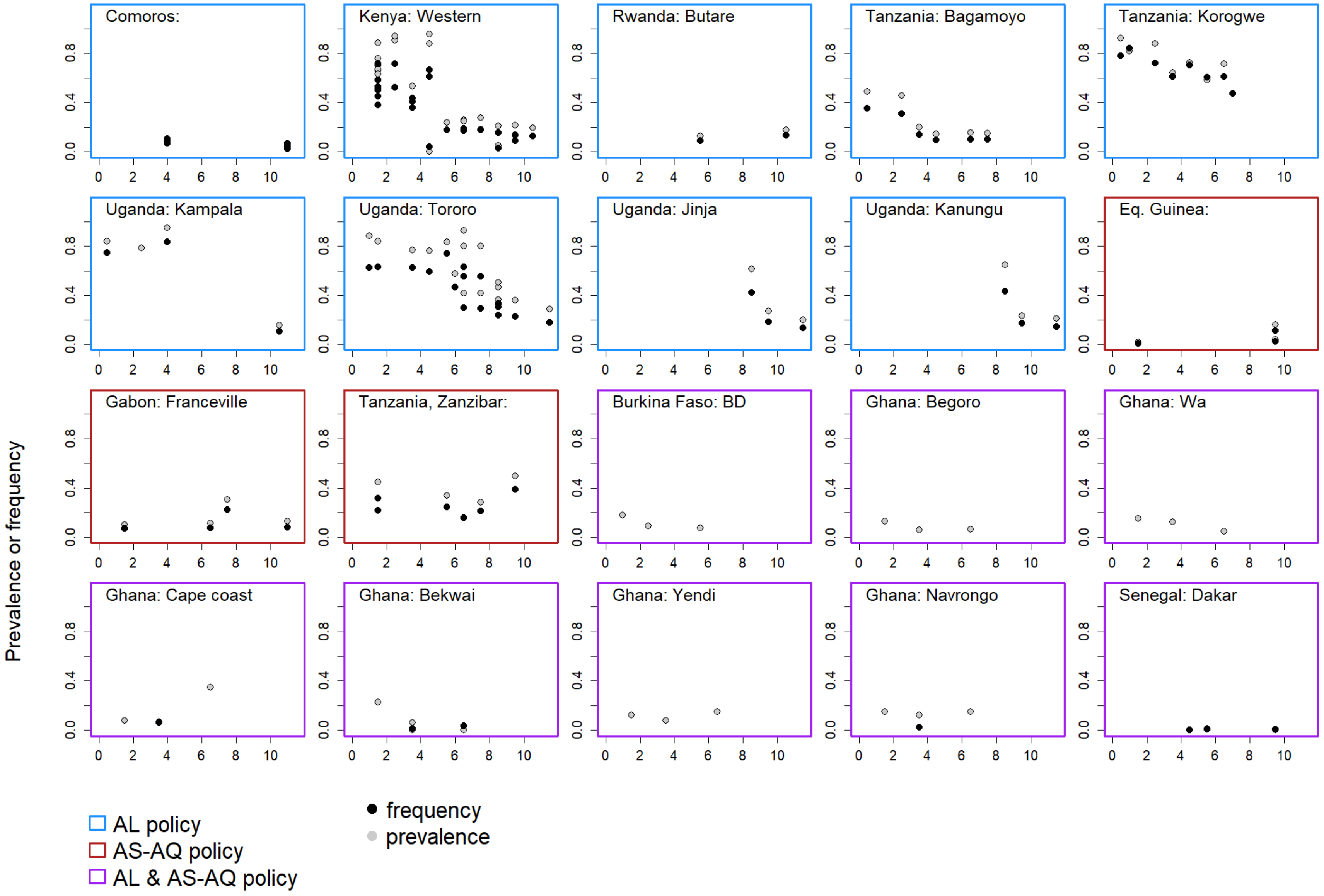

Figure S5. Rates of change of the prevalence and frequency of the 86Y mutant after introduction of ACT policy. Y axis = log(mutant/wild type prevalence) and log(mutant/wild type frequency) where available in locations which measured mutant prevalence/frequency at least twice, at least 3 years apart after the introduction of ACT policy, and with at least one non-zero observation. The slope gives the selection coefficient in each area. Where possible we fitted a slope for both frequency-based and prevalence-based measures (n=20 locations, shown here), but found this made little difference to the slope in almost all locations. Box outline colours indicate which ACT was first-line.

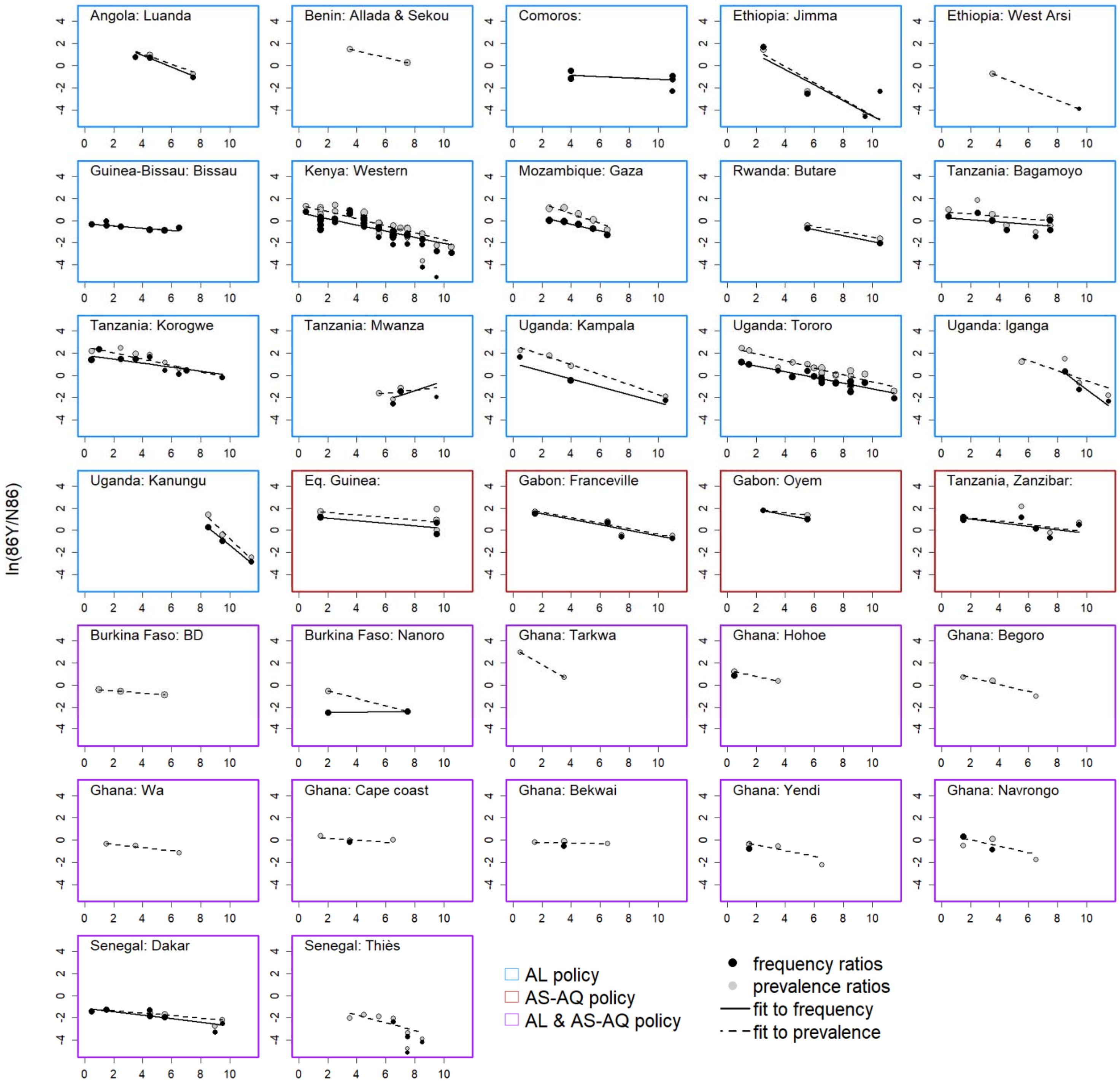

Figure S6. Rates of change of the 184F mutant after introduction of ACT policy (legend as Figure S5).

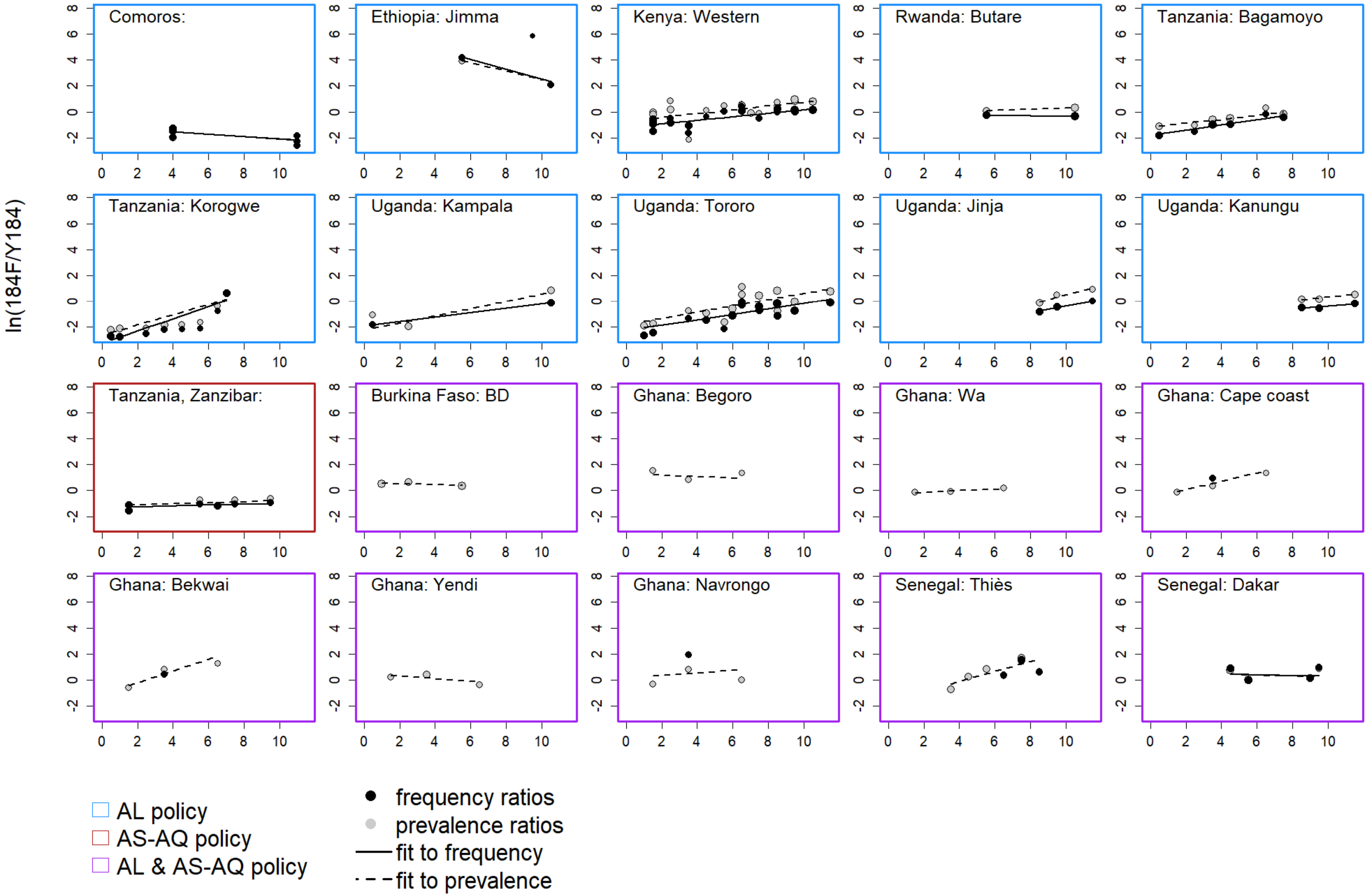

Figure S7. Rates of change of the 1246Y mutant after introduction of ACT policy (as Figure S5).

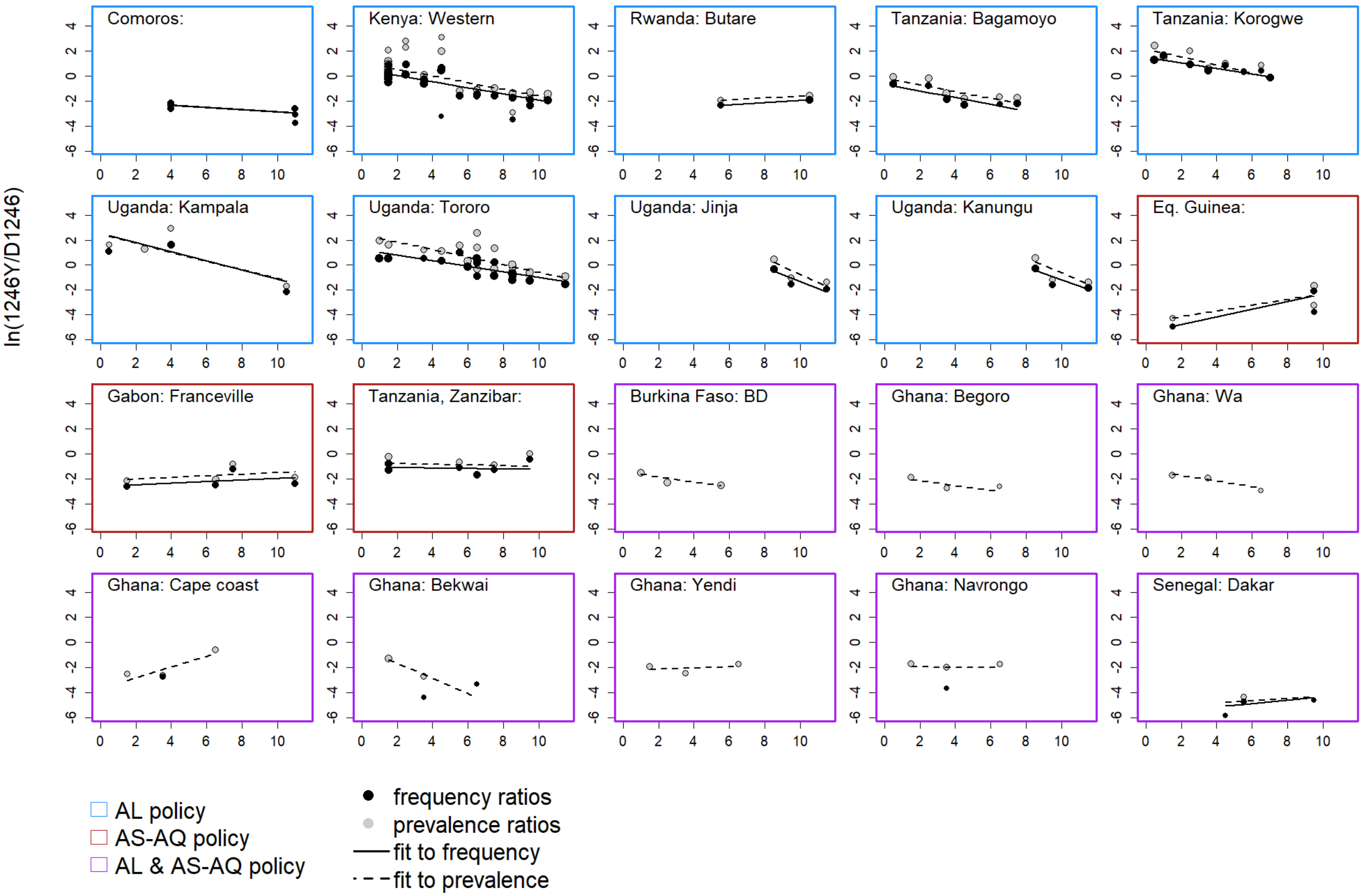

Figure S8 National first-line ACT policies in countries which also had published *Pfmdr1* data, with smaller countries/islands and their policies labelled. Predominant first-line policies were defined for each country as a policy in use for at least 5 years and lasting longer than any other ACT policy in the country. Countries coloured white are areas in which the systematic review did not identify any published *Pfmdr1* data, or which had a policy other than AL, AS-AQ or both AL and AS-AQ.

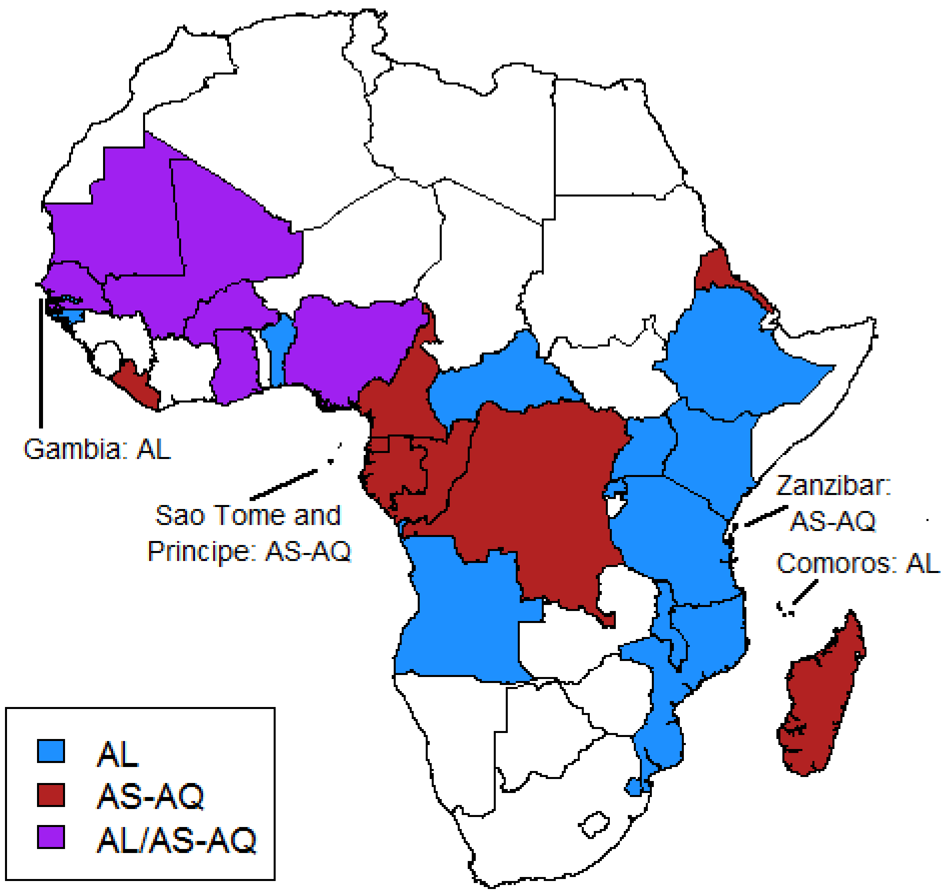

Figure S9. Associations between starting frequency/prevalence of each *Pfmdr1* mutation in a location, and its subsequent rate of change in frequency/prevalence (selection coefficient) (see Figures S2-S7 for the individual location data). All mutations show a significantly more negative change in frequency/prevalence as the starting frequency increases (p values indicated are from multilevel model analysis testing for interaction between the starting frequency of each mutation and change in frequency over time – see Methods).

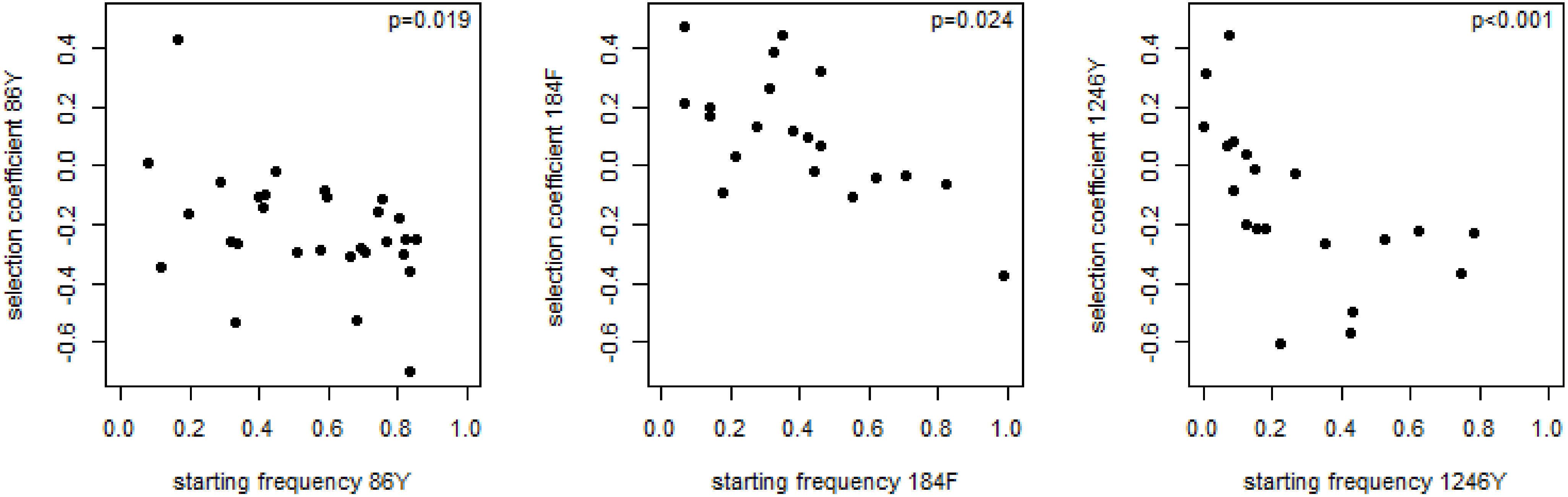

Figure S10. Trends in the 86Y mutation pre-ACT policy (year 0 = year ACT policy introduced).

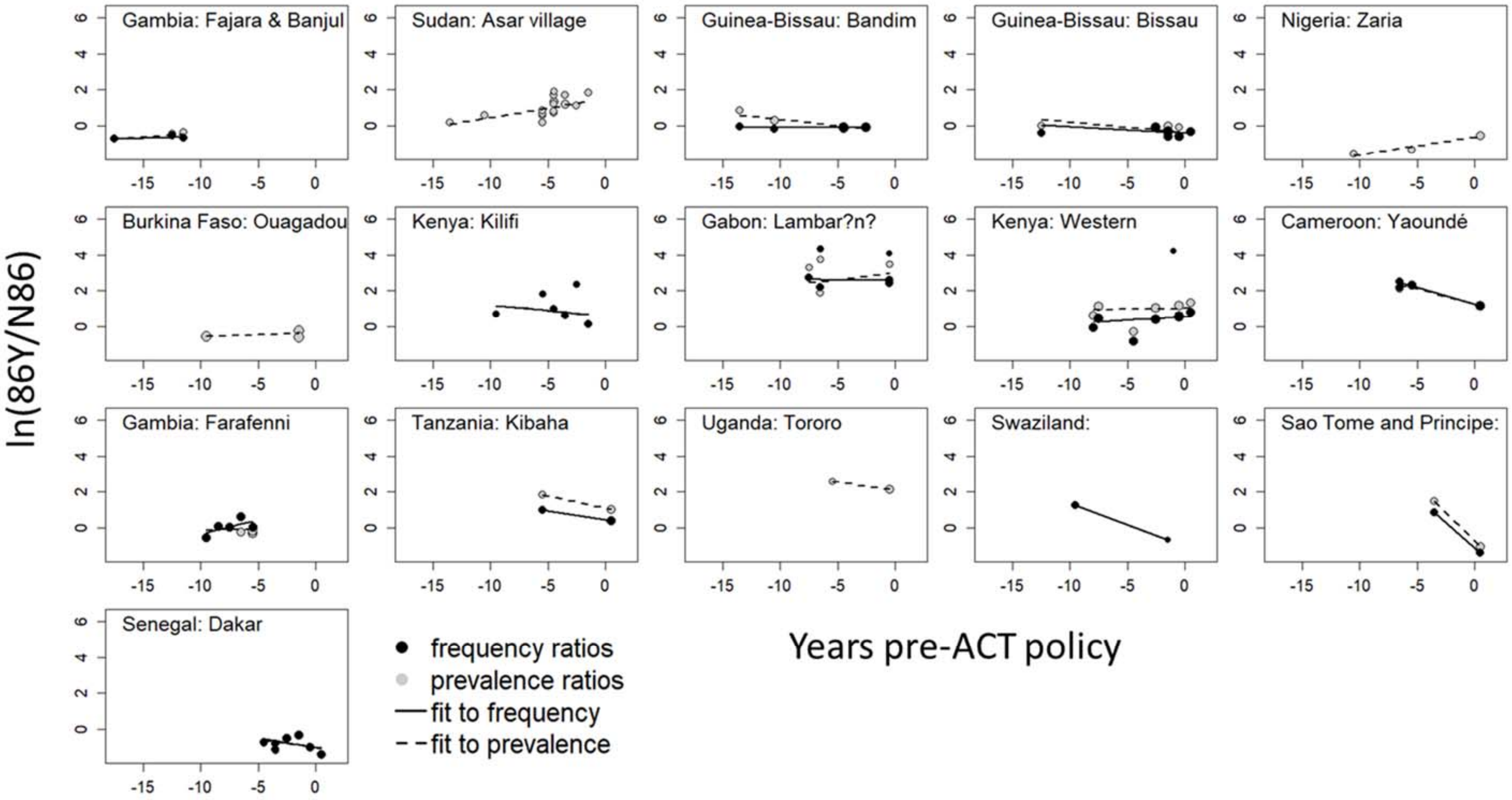

Figure S11. Trends in the 184F mutation pre-ACT policy (year 0 = year ACT policy introduced).

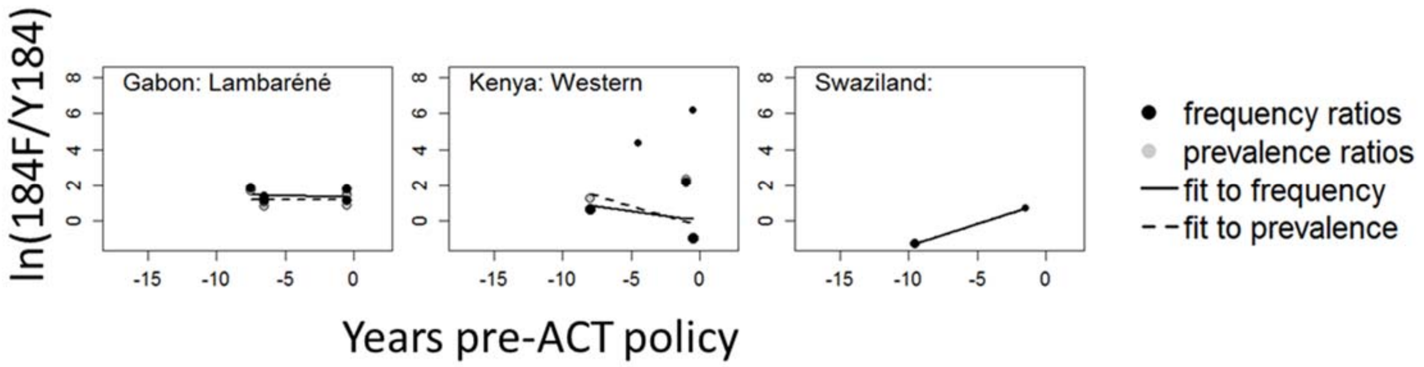

Figure S12. Trends in the 1246Y mutation pre-ACT policy (year 0 = year ACT policy introduced).

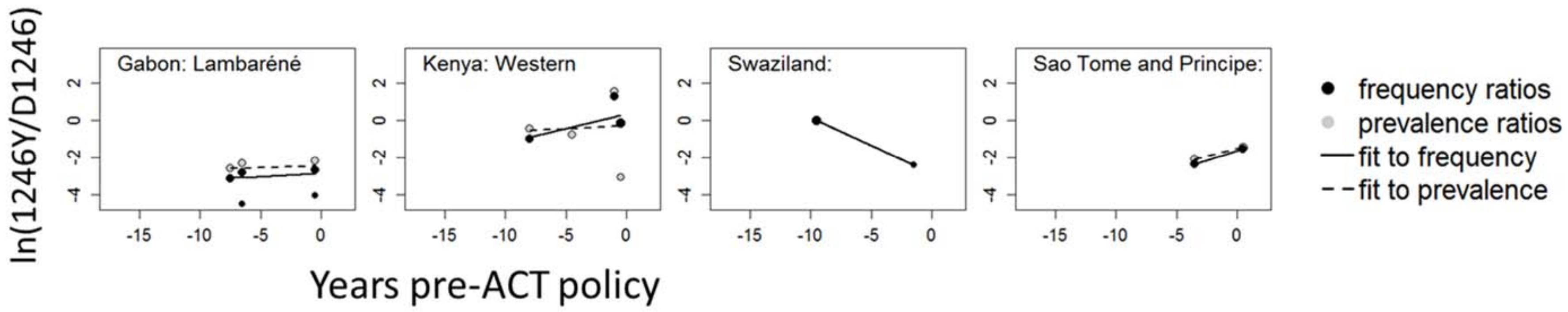

Figure S13. Frequencies of *Pfmdr1* haplotypes over time, in locations with more than one survey. The country and location are indicated in the title of each panel, followed by the main ACT policy eventually recommended (though ACT policy was not necessarily in place for the whole of the time series). Year is on the x axis, and total number of samples with haplotypes detected per survey shown above each bar.

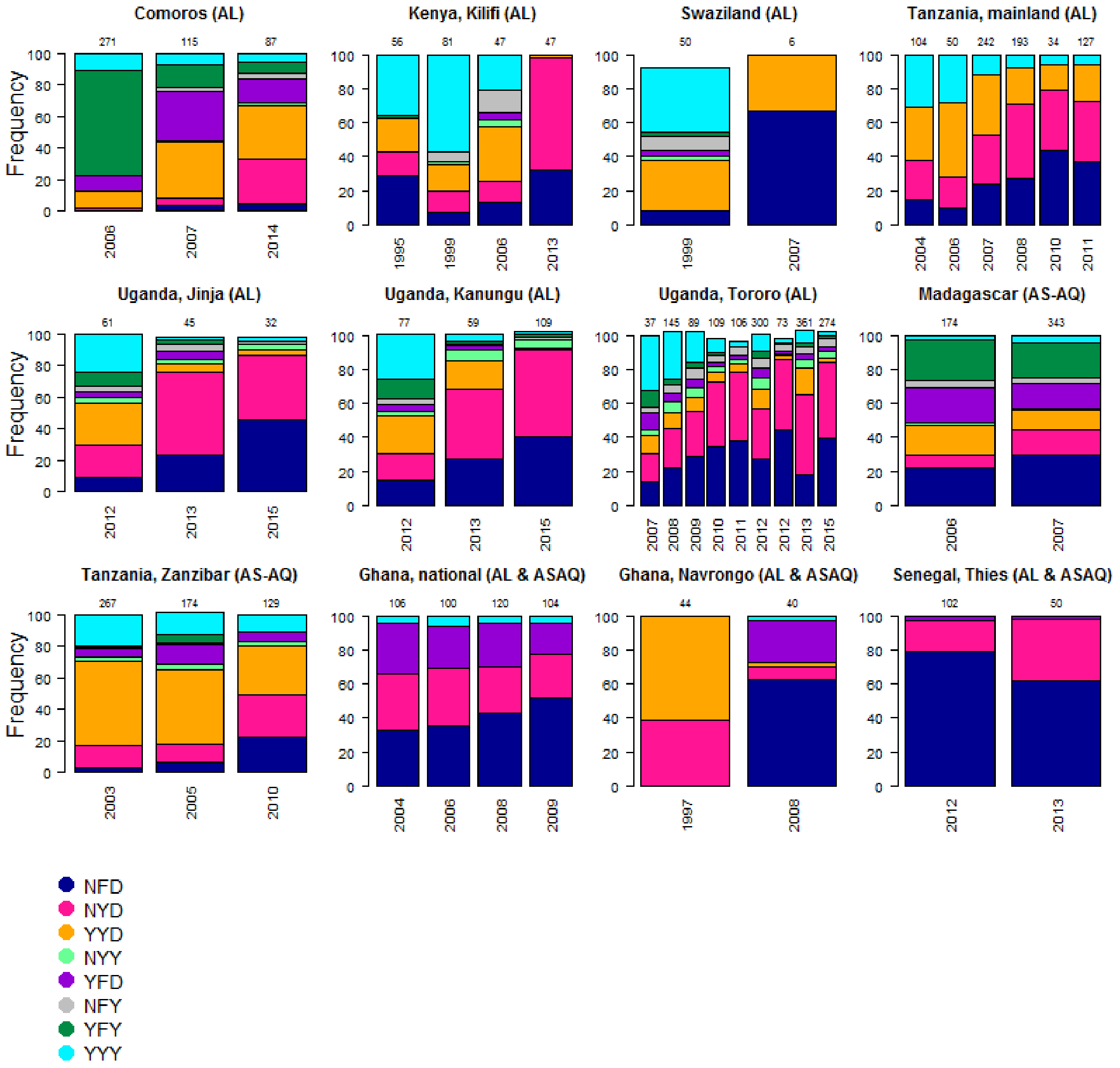

Table S1. Associations between rate of selection of mutations *Pfmdr1* 86Y, 184F, 1246Y in different locations and drug policies, drug consumption and other covariates. Associations were analysed using multi-level regression, allowing for random intercepts and random slopes by location. Regression results give the difference in selection coefficients at different covariate values, assuming 3 parasite generations per year (see methods). Two multivariate models were run separately to estimate the adjusted association between (1) ACT policy and selection and (2) between AL consumption and selection.

	86Y					184F					1246Y				
Variable	N	Unadjusted difference in selection coefficient (95% CI)	P	Adjusted difference in selection coefficient (95% CI)	P	N	Unadjusted difference in selection coefficient (95% CI)	P	Adjusted difference in selection coefficient (95% CI)	P	N	Unadjusted difference in selection coefficient (95% CI)	P	Adjusted difference in selection coefficient (95% CI)	P
**ACT policy**<br>AL<br>AS-AQ & AL/AS-AQ	111<br>61	0<br>0.031 (0.002, 0.06)	0.055	[a]<br>0<br>0.022 (-0.019, 0.064)	0.299	69<br>41	0<br>-0.029 (-0.080, 0.022)	**0.280**	[a]<br>0<br>-0.004 (-0.054, 0.046)	0.871	112	0<br>**0.074 (0.036, 0.113)**	**<0.001**	[a]<br>0<br>0.001 (-0.059, 0.062)	0.967
**Chloroquine coverage** [b]<br><20%<br>>=20%	98<br>55	0<br>-0.008 (-0.045, 0.03)	0.694	[a]<br>**0**<br>**-0.063 (-0.109, -0.017)**	**0.009**	-	-	-	-	-	76<br>27	0<br>0.021 (-0.060, 0.102)	0.611	[a]<br>0<br>-0.025 (-0.114, 0.063)	0.578
**Slide-prevalence** [c]<br><20%<br>>=20%	74<br>98	0<br>0.014 (-0.018, 0.046)	0.396	[a]<br>0<br>0.027 (-0.002, 0.057)	0.085	44<br>66	0<br>-0.003 (-0.055, 0.049)	0.910	[a]<br>0<br>0.006 (-0.041, 0.054)	0.800	38<br>74	0<br>0.010 (-0.036, 0.056)	0.691	[a]<br>0<br>-0.023 (-0.053, 0.007)	0.150
**Initial mutant frequency/ prevalence**<br>per 10% change	174	**-0.007 (-0.012, -0.001)**	**0.019**	[a]<br>**-0.013 (-0.022, -0.004)**	**0.005**	110	-0.014 (-0.025, -0.003)	**0.024**	[a]<br>-0.009 (-0.02, 0.002)	0.128	113	**-0.010 (-0.015, -0.006)**	**<0.001**	[a]<br>-0.009 (-0.017, -0.001)	0.034
**AL coverage** [d]<br><20%<br>>=20%	77<br>28	-0.008 (-0.035, 0.02)	0.592	[e]<br>-0.001 (-0.046, 0.043)	0.951	-	-	-	-	-	41<br>25	-0.017 (-0.067, 0.033)	0.570	[e]<br>0.010 (-0.026, 0.046)	0.593

a In multivariate model including ACT policy, chloroquine coverage, transmission intensity and initial mutant frequency.

b mean % of antimalarials reportedly taken by under five year olds which are chloroquine during the time period over which *Pfmdr1* data were available (DHS, MIS surveys).

c In 2-10 year olds: mean prevalence estimated from the Malaria Atlas project during the time period over which *Pfmdr1* data were available.

d mean % febrile RDT+ under five year olds receiving ACT in countries with AL policies during the time period over which *Pfmdr1* data were available (estimates from Malaria Atlas Project). Analysed in countries with AL policies only, in a separate model.

e After adjusting for chloroquine coverage, transmission intensity and initial mutant frequency (adjusted coefficients are not shown for the other variables).
